# When children can explain why they believe a claim, they suggest a better empirical test for that claim

**DOI:** 10.1098/rsos.241875

**Published:** 2024-12-11

**Authors:** Tone K. Hermansen, Kamilla F. Mathisen, Samuel Ronfard

**Affiliations:** ^1^ Department of Psychology, University of Oslo, Forskningsveien 3A, Oslo 0373, Norway; ^2^ Department of Psychology, University of Toronto Mississauga, 3359 Mississauga Rd, L5L 1C6, Mississauga, Ontario CCT 4059, Canada

**Keywords:** information seeking, uncertainty reasoning, testing claims, exploration

## Abstract

We tested the hypothesis that children’s ability to reflect on the causes of their uncertainty about a surprising claim allows them to better target their empirical investigation of that claim—and that this ability increases with age. We assigned 4–7-year-old children (*n*=174, M_age _= 68.77 months, 52.87% girls) to either a prompted or an unprompted condition. In each condition, children witnessed a series of vignettes where an adult presented a surprising claim about an object. Children were then asked whether they thought the claim was true or not, how certain or uncertain they were, and how they would test that claim. In the prompted condition, children were also asked why they were certain or uncertain. As predicted, older children were more likely to justify their beliefs and to suggest targeted empirical tests, compared with younger children. Being prompted to reflect on their uncertainty did not increase children’s ability to *generate* an efficient test for those claims. However, exploratory analyses revealed that children’s ability to provide a plausible reason for their beliefs did, controlling for their ability to *select* an efficient test for a claim. This suggests that developments in children’s reasoning about their beliefs allow them to more effectively assess those beliefs empirically.

## 1. Introduction

Across domains, children appear to become more intentional in their actions with increasing age [1]. For example, from 4 to 7 years of age, children show increased intentional control when seeking information [2,3], especially after hearing a surprising claim [4]. The current study tests the hypothesis that older children’s more targeted and efficient exploration following surprising claims may reflect a greater awareness of *why* they are sceptical about a surprising claim and that knowing *why* one is uncertain about a claim allows children to devise and implement more targeted information-seeking strategies. In doing so, the current study advances our understanding of the transition from intuitive science [5] to explicit scientific thinking and reasoning [6].

Infants and young children seek information when they are uncertain. These information-seeking behaviours are informative and facilitate belief revision [5]. For example, infants will selectively explore a train they have just seen float in mid-air rather than play with a novel toy [7], 20-month-old infants will ask for help when they are unsure where a toy is hidden [8], and 4-year-old children engage in more exploratory play when evidence is confounded rather than unconfounded [9]. These adaptive behaviours suggest an early emerging sensitivity to uncertainty that may be distinct from the ability to report on one’s uncertainty [10]. In support of this hypothesis, Lapidow *et al*. [11] found that 4- and 5-year-old children’s explicit reports of their confidence about the presence of a target shape did not differ significantly when the shape was visible, partially hidden or fully hidden. In contrast, when asked to choose which window to explore, children’s exploration differed significantly, with children most often choosing to explore the fully occluded shape. By implication, young children do not need to be able to accurately express their uncertainty in order to engage in selective exploration. However, it is possible that children’s ability to reflect on their uncertainty does influence *how* they seek information. That is, being able to reflect on one’s uncertainty may predict more efficient search.

Improvement in children’s metacognitive abilities—the ability to be aware of and contemplate thinking [12]—may allow children to improve the efficiency and effectiveness of their exploration. Indeed, with increasing age, children improve in their judgements and reasoning about their own uncertainty [13] alongside the efficiency of their exploration [14] and their reasoning about how to gather information [15]. Between 3 and 7 years old, children’s uncertainty judgements become better calibrated [13,16], their ability to reason about uncertain causes improves [17,18], and they are increasingly able to reflect on the relation between beliefs and evidence [19]. During the same time, children’s exploration becomes increasingly attuned to the opportunities and constraints of a situation. For example, from 3 to 9 years old, children’s exploratory search strategies become increasingly targeted to the task [3,14], they also improve in their ability to identify the most informative questions [20,21] and become more likely to exploit the environment to gather the information they need [2]. Finally, children’s ability to reason about how to test a claim improves. Between 4 and 8 years old children increasingly come up with relevant exploration strategies when explicitly asked to do so [22] and improve their ability to generate and test hypotheses using unconfounded experimental designs [23–27].

These data suggest an important role for children’s metacognitive abilities for *how* they search for information. However, these data do not yet provide direct evidence for their connection because none of these prior studies collected information about children’s metacognitive abilities. Thus, there is no direct evidence for a correlation between increased metacognition and improved search efficiency. The current study seeks to empirically test this connection by examining links between children’s reasoning about their own uncertainty and their suggestions for whether and how to test a surprising claim. In a set of recent studies, researchers have investigated young children’s exploratory behaviours following a surprising claim, finding that their exploration is generally informative and can lead to knowledge updating [4,28–31]. Moreover, consistent with the above-referenced research on children’s exploration following their *observation* of surprising phenomena, children’s exploration following surprising *claims* becomes increasingly targeted and efficient. For example, in past research, 4- to 7-year-old children were presented with a set of five Russian dolls. Children were asked which of the dolls was the heaviest. Consistent with prior research demonstrating that even infants associate size with weight [32], all children replied that the biggest doll was the heaviest. Children were then assigned to one of two conditions to have that intuition either confirmed or contradicted. When their intuitions were contradicted, they were told that the smallest doll rather than the biggest doll was the heaviest. Children were then left alone with the dolls. Compared with children whose intuitions were confirmed, children whose intuitions were contradicted selectively explored the dolls: on average, they were more likely to pick up the smallest doll and the biggest doll. However, only older children (6- and 7-year-olds) engaged in targeted testing of the surprising claim by picking up the biggest and the smallest doll *at the same time*, a direct test of the surprising claim [31]. This age change in the efficiency of children’s investigation of a surprising claim was recently replicated on a third-party task where 4- to 8-year-old children were asked to reason about how another child should act after hearing a set of eight surprising claims. These claims targeted different object properties and varied in whether they were simple claims, e.g. this (the smallest) object is very heavy or comparative claims, e.g. this (the smallest) object is the heaviest (compared with much larger objects). Results revealed that with increasing age children made more targeted exploration recommendations—for example, suggesting that the child pick up only the target object for simple claims and suggesting that the child pick up the smallest and the biggest object for the comparative claims. With increasing age, children were also more likely to justify such exploration decisions by expressing uncertainty about the truth of the surprising claim. Such reports of uncertainty were associated with children’s suggestion of more targeted testing, controlling for age [28].

Why might children’s report of their uncertainty about a surprising claim be associated with more efficient and targeted recommendations for testing that claim? Children’s report of their uncertainty about the surprising claim in this prior study could reflect two distinct aspects of uncertainty: (i) the ability to recognize and accurately report feelings of uncertainty (e.g. ‘I am not sure what to believe’), and (ii) the ability to introspect on the *causes* of that uncertainty, as reflected in the ability to provide an explanation for why the adult’s claim is unlikely to be true (e.g. ‘I am not sure what to believe, because what they said conflicts with my previous experience’). These two aspects are distinct because being uncertain about a claim does not tell you about why you feel this way [10]. Between the ages of 3 and 7 years old, children become increasingly able to recognize feelings of uncertainty as reflected in their report on their own ignorance and uncertainty [13], with children from the age of 5 evincing increasingly precise reasoning about their own knowledge [16]. During the same time period, children also become better able to engage in diagnostic reasoning about uncertain causes, with older children providing more correct assessments [18] and being less sensitive to task constraints [33]. Diagnostic reasoning is the ability to identify the cause of a phenomenon among a set of possibilities [17]. Such reasoning is similar to being able to identify the causes of one’s uncertainty about a claim, as identifying the source of one’s uncertainty requires deducing the most likely explanation for why the claim is wrong based on a comparison of the evidence supporting prior beliefs as well as the evidence for a surprising claim. Being able to identify the reasons for one’s scepticism about a claim is likely to be a strong predictor of one’s ability to design an informative test of that claim, given that explanations drive children’s exploratory behaviours (see [34] for a review). Thus, we make four main predictions: (1) with increasing age, children will express more uncertainty about the possibility of a surprising claim; (2) with increasing age, children will be more likely to provide a plausible reason for their uncertainty about the possibility of a surprising claim; (3) with increasing age, children will be more likely to suggest targeted empirical tests for a claim—tests that provide the needed evidence to confirm or disconfirm the truth of a claim; (4) prompting children to reflect on the causes of their uncertainty about a claim will increase the likelihood that they generate an efficient test of that claim. This effect of prompting is expected to be stronger among younger children.

**Figure 1.**
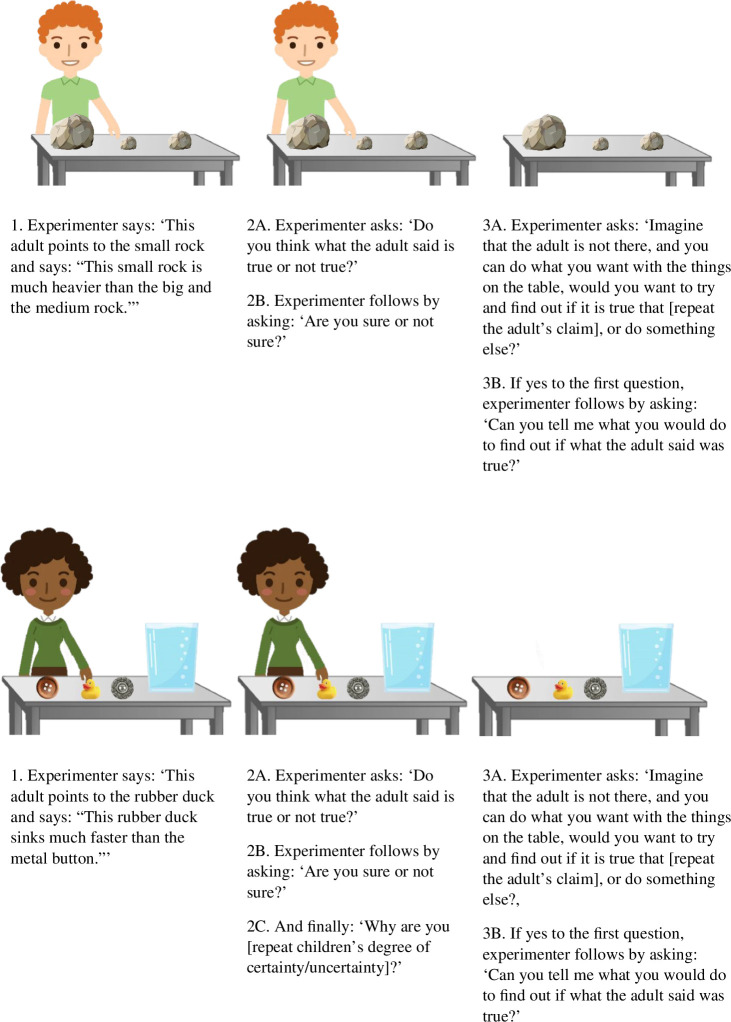
Examples of experimental procedure in unprompted (top) and prompted (bottom) condition, showing two different comparative claims: one comparing the target object to all the other objects (top) and the other comparing the target object to one of the other objects (bottom).

To test these hypotheses, we assigned children to two conditions: a prompted and an unprompted condition (see figure 1 for an illustration of a single trial). In each condition, children were presented with four surprising claims. For example, they were shown pictures of three rocks of increasing size. They then heard a speaker say that the smallest rock is much heavier than the other rocks before being asked whether they think that this is true or not true (Belief question) and whether they are sure or not sure about this (Uncertainty question). Then, in the prompted condition only, children were asked why they felt this way (Reasoning question). These responses were coded for the presence or absence of a plausible explanation (i.e. including a description of a mechanism or regularity), saying for example, that ‘bigger objects are typically heavier than smaller objects’. Finally, for both conditions, children were asked whether they thought it might be worthwhile to try and determine whether the adult’s claim was true or not (Explore question), and if so, how they would proceed (Design question). Children’s responses were coded based on whether children’s exploration suggestions would provide evidence that the adult’s claim is true, and whether they did so inefficiently (e.g. they suggested gathering either too little or too much data) or efficiently (e.g. they suggested gathering enough data and the right type of data). In the preceding example, this would mean comparing the weight of all of the rocks, which is the only way to know if the smallest rock is the heaviest of them all.

To test our four hypotheses, we conducted the following confirmatory analyses. We assessed prediction (1) by combining the certainty response of children across both conditions and testing whether children express more uncertainty with age. Prediction (2) was tested by looking at whether older children in the prompted condition provided more plausible reasons for their scepticism than younger children. Prediction (3) was tested by looking at whether older children provide more targeted empirical tests than younger children, and that this effect also held when controlling for their ability to identify an efficient test when provided with multiple options. Prediction (4) was assessed by testing whether children in the prompted condition were more likely to provide a more targeted empirical test than children in the unprompted condition.

Of course, children’s awareness of the uncertainty of a claim may not be sufficient for children to engage in targeted testing of that claim. Children’s ability to design an effective test will determine whether the necessary information can be sought. Thus, at the end of the experiment, we presented children with three additional surprising claims, this time with three options for assessing the truth of those claims. Children were asked to select the alternative that best enables them to test whether the adult’s claim is true or not (Select question). Given prior work showing that 4-year-old children are able to select the most informative test to understand a causal system [35] and to select the most informative question when presented with multiple options [21], our final prediction (5) was that we expect all children in our study to do so. However, this additional test was important to rule out the possibility that age-related differences in the type of empirical test suggested by older children do not simply reflect age-related differences in knowledge of testing strategies. Furthermore, it allowed us the opportunity to control for age-related differences in children’s capacity to verbally formulate testing strategies.

## 2. Methods

### 2.1. Participants

The final sample consisted of 174 4- to 7-year-old children (M_age_ = 68.77 months, range = 47–95 months, 92 girls (52.87%), 82 boys (47.13%)). An additional (*n* = 5) children were tested but were excluded from the final analyses due to the following: (i) technical (*n* = 1), (ii) experimenter error (*n* = 2), (iii) age (*n* = 1), and (iv) participated in the pilot (*n* = 1). To enable a diverse sample from rural and urban areas, participants were recruited through childcare centres across the country, as well as through social media channels, with half of the final sample coming from the central eastern region (*n* = 87, 50%) and the others coming from the rest of the country (i.e. representing the southern, western, inland and northern regions (*n* = 78, 44.83%)) (missing: *n* = 9, 5.17%). The majority of children had Norwegian only as their mother tongue (*n* = 136, 78.16%), while some had Norwegian and another language as their mother tongue (*n* = 27, 15.52%), and only a few reported not having Norwegian as their mother tongue, although attending daycare and school in Norwegian and having one parent speaking both Norwegian and another language (*n* = 2, 1.15%) (missing: *n* = 9, 5.17%). In terms of education, 82.91% (*n* = 146) of the participating parents reported that either they or their partner had completed a BA degree or higher (missing: *n* = 9, 5.17%), and 75.29% (*n* = 131) of the households reported an average income above the median Norwegian income (*ca* 60 000 USD, Statistics Norway, 2023) (missing: *n* = 10, 5.75%)—thus reflecting typical middle-class households in Norway. Informed consent was obtained from the child’s parents in advance of testing.

We based our sampling plan on a power analysis, using effect size information from previous studies with a similar design and age range [30,31]. Using GPower 3.1 [36], this analysis revealed that to detect a low-medium to medium effect (*f* ^2^ = 0.08–0.15), with 80% power at alpha = 0.05, the analyses associated with the most resource-demanding analysis would require a sample of 175 participants.

### 2.2. Ethics and data handling

The overall project was approved by the internal ethical research committee (Department of Psychology, University of Oslo, no.: 16842024) and the local authorities on data protection (NSD, no.: 843823), and supported by a departmental research grant to the first author from the Department of Psychology, University of Oslo. Person-identifying video recordings from the testing session are stored in the university’s internal, secure storage system TSD, accessible only to the two first authors and research assistants associated with the two first authors. Anonymized data supporting the findings of the study are available in the Open Science Framework at [37], together with study stimuli and analysis scripts. The manuscript was accepted following stages 1 and 2 reviews at Peer Community In Registered Reports and recommendations can be viewed at [38].

### 2.3. Procedure

Parents agreeing to take part in the study with their child were invited to perform the tasks through an online web portal at a time of their convenience. One week prior to their scheduled participation, parents received instructions as to the procedures of the main testing session. On the day of testing, parents were asked to log onto the online platform together with their child, from which the experimenter introduced the Familiarization task, the Experimental task and the Selection task. Finally, parents received a short online questionnaire on demographic background information. The session was video recorded for later coding.

A *Familiarization task* was presented to all children prior to starting the main experiment. We used this task to inform children about what was about to happen (i.e. hear a set of claims and be asked a set of questions about what they believe and how certain they are), and that they now had the opportunity to practise two times first.

For the *Experimental task*, all children were assigned to one of two conditions of four trials: a prompted and an unprompted condition. In each condition, an adult informant presented children with a surprising claim about an object in front of them on the screen, following which the experimenter asked the child: (i) whether they believed the claim or not (Belief question), and (ii) how certain or uncertain they were in their belief (Uncertainty question). In addition, children in the prompted condition only were asked to reflect on *why* they felt the way they did about their belief (Reasoning question). Finally, children in both conditions were asked whether they wanted to figure out whether the adult’s claim was true or not (Explore question), and if so, how they would go about doing so (Design question). The type of trial and placement of the referent object was counterbalanced across trials and participants.

To conclude the session, children were presented with the *Selection task* to assess their ability to identify an effective test. In this task, children were again presented with a series of three surprising claims, but rather than asking children whether they wanted to find out the truth about the claim and if so how, they were simply asked to select between a set of three pre-specified options as to how they might go about testing the claim. The options varied in the degree to which the claim would be sufficiently tested, allowing children to select between manipulating the referent object, the referent object and one of the alternative objects, or the referent object and both the alternative objects. The order of the action options was counterbalanced across trials and participants. To ensure that the trials used in the selection task were not perceived differently to the trials used in the experimental task, all trials for both tasks were randomly selected from the same pool of trials.

### 2.4. Familiarization task

During the familiarization task, children were presented with two trials containing a familiar object, a blue ball and a yellow box and faced with a claim that was either easy to be sure of (e.g. that a blue ball is blue), or something to be less sure of (e.g. that a yellow box is pink) (for details see Appendix 1 under Materials in [37]). In the first trial, children were presented with a claim about an object’s which matched its visible properties: ‘Lets imagine that you see a picture of a ball—like this one—and I tell you that it is blue (a blue ball is shown on the screen). Do you think it is true or not true that this ball is blue?’ To guide the child in how to respond, the informant followed up by saying: ‘If you think it is true, you can click on the green button, and if you think it is not true you can click on the red button.’ After the child responded, the experimenter went on to ask the child: ‘Are you sure or not sure?’ Again, to guide the child in how to respond, the informant followed up by saying: ‘If you are sure, you can click on the green button, and if you are not sure you can click on the red button.’ In the second trial, children were presented with a claim about an object which did not match its visible properties: ‘Lets imagine that you see a picture of a box—like this one—and I tell you that it is pink (a yellow box is shown on the screen). Do you think it is true or not true that this box is pink?’ Followed by the same instructions on how to respond as in trial one. After the child responded, the experimenter went on to ask the child: ‘Are you sure or not sure?’ Followed by instructions on how to respond. Finally, to prepare children to provide a response to an open-ended question, they were asked: ‘Can you tell me, what do you think could be inside this box?’ If the child wanted either of the questions repeated, the experimenter repeated the question. The order of response options was counterbalanced across participants.

### 2.5. Experimental task

In each trial of the main experiment, children were presented with a scenario in which an adult informant presented a surprising claim about one of three objects visible on screen followed by a set of questions (inspired by [4]). For example, presenting a claim that runs counter to what children typically think about the relative weight of small objects as compared with big objects, the adult informant referred to a small rock next to two larger rocks saying: ‘This small rock is much heavier than the big and the medium rock’. Considering previous work showing that young children are sensitive to an informant’s knowledge [39], the claim was presented without any reference to knowledgeability. After hearing the informant’s claim, the experimenter first asked the child the Belief question: ‘Do you think what the adult said was true or not true?’ before asking the child the Uncertainty question: ‘Are you sure or not sure?’. Next, children in the prompted condition were asked the Reasoning question: ‘Why are you [repeat children’s degree of certainty/uncertainty]?’. Finally, children in both conditions were asked the Exploration question: ‘Imagine that the adult is not there, and you can do what you want with the things on the table, would you want to try and find out if it is true or not true that the small rock is heavier than the big and the medium rock, or do something else?’. Following a confirming response to this question, they were asked the Design question: ‘Can you tell me what you would do to find out if what the adult said was true or not true?’. Across all trials, if children wanted either of the questions repeated, the experimenter repeated the question. Illustrations of a trial from each of the two conditions are presented in figure 1. Note that each child was presented with a selection of different claims across the trials, with half of the children presented with one set of trials and the other half presented with a different set of trials (in §3 labelled as ‘Blocks’), and figure 1 is only meant as an example of the general experimental procedure and to highlight the key difference between the two conditions (for a full overview of the trial types and blocks, see Appendix 1 under Materials [37]).

### 2.6. Selection task

To control for individual differences in children’s ability to identify an efficient test of a surprising claim, children were presented with three additional surprising claims along with three options for assessing the truth of those claims and asked to select which option they would use to find out if what the adult said is true. Thus, rather than generating their own action suggestions, they were asked to choose between three options that reflected inefficient non-comparative exploration (1), inefficient comparative exploration (2), or efficient exploration (3) (for details, see Appendix 1 under Materials [37]). For example, following the adult’s claim that ‘This small rock is much heavier than the big and the medium rock’, children were asked: ‘What would you do to find out whether it is true that this little rock is much heavier than the big and the medium rock?’, immediately followed by the three alternative response options: ‘Lift the little rock (1), Lift the large, the small and the middle rock (2), or Lift the large and the small rock (3)?’. Across all trials, if children wanted either of the questions repeated, the experimenter repeated the question.

### 2.7. Data processing

#### 2.7.1. Coding

Video recordings from the three tasks were coded post-testing according to the below categories for the different questions by the second author and a research assistant blind to the study’s hypotheses, with one coding all videos and the other coding *ca* 20% to allow for reliability estimation. Reliability was assessed as percentage agreement, with agreement above 75% on each item considered acceptable. To meet criteria for both the Reasoning and Design questions, coding reliability was estimated based on the responses from children in the prompted condition who indicated that they wanted to find out if the claim was true or not (*n* = 52, 57% of sample in prompted condition and 29% of total sample). For the Reasoning question, this revealed an average of 84.53% agreement across the four experimental trials (T_1–4_) (average Cohen’s Kappa = 0.723) (T_1_ = 78.8%, *K* = 0.619; T_2_ = 88.2%, *K* = 0.780; T_3_ = 86.5%, *K* = 0.769; T_4_ = 84.6%, *K* = 0.725). For the Design question, there was approximately 90.85% agreement across the four trials (average Cohen’s Kappa = 0.860) (T_1_ = 92.3%, *K* = 0.897; T_2_ = 94.2%, *K* = 0.907; T_3_ = 94.2%, *K* = 0.906; T_4_ = 82.7%, *K* = 0.733). In the event of coder discrepancies, differences due to coding errors were corrected, while discrepancies due to coder disagreement were resolved through discussion with the first author.

#### 2.7.2. Belief question

Children’s responses to the Belief question were coded based on whether they said they thought the claim was true (0) or not true (1). A lack of response was coded as missing (N_per trial_: T_1_ = 1, T_2_ = 2, T_3_ = 2 and T_4_ = 2). Excluding responses coded as missing, we created an average score to reflect children’s propensity to believe the surprising claim.

#### 2.7.3. Uncertainty question

Children’s responses to the Uncertainty question were coded based on whether they were sure (0) or not sure (1) in their beliefs. A lack of response was coded as missing (N_per trial_: T_1_ = 2, T_2_ = 2, T_3_ = 2 and T_4_ = 3). Excluding responses coded as missing, an average score was created based on the four trials in the Experimental task.

#### 2.7.4. Reasoning question

We coded children’s spontaneous responses to the Reasoning question into two broad categories depending on whether they provided a response that reflected the absence (0) or presence (1) of a plausible explanation. That is, a response was considered a plausible reason if including a description of a mechanism (e.g. saying ‘some things can be filled with stuff’) or statistical regularity (e.g. saying ‘bigger things are often heavier’) relating to the objects at hand, while responses that did not include a reference to such characteristics (e.g. saying ‘maybe it just is’ or ‘I don’t know’) were considered lacking a plausible explanation. A lack of response was coded as missing (N_per trial_: T_1_ = 7, T_2_ = 7, T_3_ = 11 and T_4_ = 10). For children in the prompted condition, and excluding responses coded as missing, an average score was created based on the four trials in the experimental task. For children in the unprompted condition, this average score was coded as missing.

#### 2.7.5. Exploration question

Children’s responses to the exploration question were coded based on whether they said they did not want to find out the truth of the claim or simply wanted to do something else (0) or whether they said they wanted to find out if the claim was true (1). Responses that were not relevant for the question were coded as 2, but discarded from the main analyses. A lack of response was coded as missing (N_per trial_: T_1_ = 9, T_2_ = 5, T_3_ = 3 and T_4_ = 7). Excluding responses coded as missing, an average score was created based on the four trials in the Experimental task.

#### 2.7.6. Design question

Children’s spontaneous responses to the Design question were first coded into three broad categories depending on whether they provided a response that reflected inefficient non-comparative (1), inefficient comparative (2) or efficient (3) exploratory behaviours. Efficient exploration included descriptions of a comparative manipulation of all relevant objects only (e.g. lifting all rocks to assess which is the heaviest of them all, or dropping the rubber duck and the metal button into the water container to assess which of the two sinks faster). Inefficient relevant exploration encompassed responses that are comparative but reflect too much exploration (e.g. exploring all objects, when the claim relates to only two of the objects) or too little exploration (e.g. exploring two of the objects when all three objects require assessment), and inefficient irrelevant exploration involved only suggestions to explore a single key referent object when a comparative assessment is required. Children who suggested irrelevant exploratory behaviours unrelated to the claim or to testing it (e.g. suggesting to smell a stone, or saying that ‘but it is not true’) or who expressed that they did not know were coded as 0. A lack of response was coded as missing (N_per trial_: T_1_ = 56, T_2_ = 68, T_3_ = 69 and T_4_ = 65). For the purpose of the main planned analysis, we then recorded the design variable by focusing on the contrast between children’s suggestions of inefficient (0) versus efficient exploration (1), collapsing across the inefficiency categories. We then first created a sum score reflecting the total number of times children suggest an efficient exploration strategy (hereafter called *DesignSum*) as well as an average score (hereafter called *DesignAverage*) based on the number of trials children suggest to explore, excluding children who only suggested behaviours not related to testing the claim (*n* = 56), never responded (*n* = 8) or never wanted to explore the claim (*n* = 43). However, given that this excluded a large number of children who wanted to explore the claim but never suggested testing it, we created a new variable to use for exploratory analyses, now distinguishing between whether they wanted to find out the truth of the claim and: never suggested a test (i), suggested only inefficient testing strategies (ii), or suggested an efficient testing strategy on one or more occasions (iii) (hereafter called *DesignAny*). We also created a final variable including also children who reported that they did not wish to find out the truth about the claim (coded as 0), hereafter called *DesignAll*.

#### 2.7.7. Selection question

Children’s responses to the selection question were first coded in a similar vein as their responses to the Design question, and based on whether they selected the option reflecting inefficient non-comparative (i), inefficient comparative (ii), or efficient (iii) exploratory behaviours. A lack of response was coded as missing (N_per trial_: T_1_ = 11, T_2_ = 10 and T_3_ = 11). For the purpose of the main analysis, an average score was then created based on the three trials in the selection task, excluding responses coded as missing.

#### 2.7.8. Exclusion

To be considered eligible for inclusion in the main analysis, children were required to provide at least one response to a question where response options are readily available (i.e. Belief question, Uncertainty question, Explore question and Selection question). A complete lack of response on these questions was considered a failure to comply with the task. A lack of responses to a trial without such response options (i.e. Reasoning question or Design question) was not considered a failure to comply with the task, as some children were expected to struggle to spontaneously generate a response for these questions. We excluded all cases where there is a disruption of the trial due to technical (*n* = 1) or experimenter error (*n* = 2). In the end, we also had to exclude one child due to age (*n* = 1), and one because we noticed post hoc that they had participated in the pilot (*n* = 1).

## 3. Results

### 3.1. Do children express greater scepticism towards a surprising claim with increasing age?

**Figure 2.**
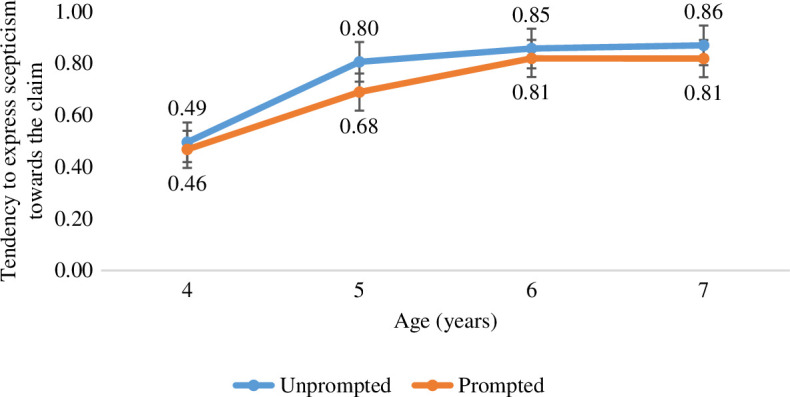
Children’s tendency to express scepticism towards the claim, as reflected in the average Belief score across the four trials (0 = always true, 1 = never true), for each age group and condition. Error bars reflect standard error.

As a preliminary assessment, we explored the extent to which children believe a surprising claim to be true or not, and whether this changed with increasing age. Overall, the majority of children expressed scepticism towards the claim on the majority of trials (T_1_ = 101, 58.38%; T_2_ = 117, 68.20%; T_3_ = 141, 81.98% and T_4_ = 127, 73.88%). However, as illustrated in figure 2, and supported by a linear regression with children’s Belief score as the dependent variable and Age (in years) as the independent variable, children were more likely to express scepticism with increasing age, *F*(1,172) = 32.12, *p* < 0.001, *R^2^* = 0.157, *b* = 0.120 (s.e. = 0.02).

Control analyses revealed no effect of Condition, *F*(1,172) = 1.07, *p* = 0.302, *R* ^2^ = 0.006, *b* = −0.051 (s.e. = 0.05); Block, *F*(1,172) = 2.80, *p* = 0.096, *R* ^2^ = 0.016, *b* = −0.082 (s.e. = 0.05); Order, *F*(1,172) = 2.59, *p* = 0.109, *R* ^2^ = 0.015, *b* = −0.079 (s.e. = 0.05); or Gender, *F*(1,172) = 0.50, *p* = 0.479, *R* ^2^ = 0.003, *b* = 0.035 (s.e. = 0.05). Furthermore, Age always remained a significant predictor of children’s tendency to express scepticism towards the belief when these variables were included as control variables in the model (all *p*s < 0.05) and there were no significant interactions (all *p*s > 0.05).

### 3.2. Do children express more uncertainty about the possibility of a surprising claim with increasing age?

**Figure 3.**
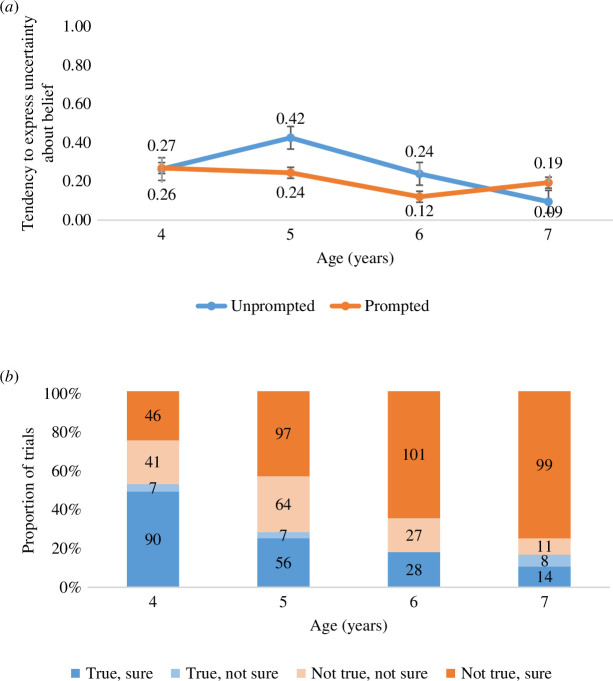
(*a*) Children’s tendency to express uncertainty about their belief, as reflected in their average uncertainty score across the four trials (0 = Always certain, 1 = Never certain), for each age group and condition. Error bars reflect standard error. (*b*) Children’s expressed certainty versus uncertainty about their relative belief in a claim, across the four age groups and collapsed across the four trials. Numbers inside the bars reflect the total number of trials children could be categorized as either believing a claim is: (1) True and sure about it (dark blue bar), (2) True, but not sure (light blue bar), (3) Not true, but not sure (light orange bar), or (4) Not true and sure (dark orange bar).

When asked how certain they were in their beliefs, and contrary to our prediction for Hypothesis 1, children expressed *less* and not more uncertainty about their beliefs with increasing age. As illustrated in figure 3*a* and supported by a linear regression with children's Uncertainty score as the dependent variable and Age (in years) as the independent variable, children were more likely to say that they were ‘sure’ about their beliefs with increasing Age, *F*(1,172) = 4.85, *p* = 0.029, *R* ^2^ = 0.027, *b* = −0.051 (s.e. = 0.02).

Control analyses revealed no effect of Condition, *F*(1,172) = 1.75, *p* = 0.188, *R* ^2^ = 0.010, *b* = −0.071 (s.e. = 0.02); Block, *F*(1,172) = 3.27, *p* = 0.072, *R* ^2^ = 0.019, *b* = −0.090 (s.e. = 0.05); or Gender, *F*(1,172) = 0.15, *p* = 0.703, *R* ^2^ < 0.001, *b* = −0.020 (s.e. = 0.05), and including these control variables did not change the main model described above; age always remained a significant predictor of children’s (un)certainty about their beliefs (all *p*s < 0.05) and there were no interactions (all *p*s > 0.05). There was, however, an effect of Order, *F*(1,172) = 5.20, *p* = 0.024, *R* ^2^ = 0.029, *b* = 0.112 (s.e. = 0.05), with children being more likely to express uncertainty if this was provided as the first of the two response options (i.e. when asked: ‘Are you “not sure” or “sure”?’, compared with when asked: ‘Are you “sure” or “not sure”?’). Although again, the effect of Age remained significant also when introducing order in a preliminary step in the model (*p* < 0.05), and there was no Order by Age interaction (*p* > 0.05).

Interestingly exploratory analyses revealed that when introducing Age in the first step of the model and adding Belief and their interaction term in the second and third step, there was a significant Age by Belief interaction, *F*(3,170) = 14.81, *p* < 0.001, *R* ^2^ = 0.207, *b* = −0.408 (s.e. = 0.07). As illustrated in figure 3*b*, separate post hoc analyses of the effect of Belief on Uncertainty within each of the four age groups, revealed that 7-year-olds appeared more likely to express certainty about their beliefs when they had first signalled that they were sceptical of the claim, *F*(1,31) = 18.97, *p* < 0.001, *R* ^2^ = 0.380, *b* = −0.817 (s.e. = 0.19). For younger children the pattern appeared reversed, with 4-year-olds being more likely to express uncertainty when they had first signalled scepticism towards the claim, *F*(1,44) = 27.48, *p* < 0.001, *R* ^2^ = 0.384, *b* = 0.553 (s.e. = 0.11). Although β-values signalled that 5-year-olds resembled 4-year-olds in their responses and that 6-year-olds resembled 7-year-olds in their responses, there was no significant effect of Belief on Uncertainty among 5-year-olds, *F*(1,54) = 1.87, *p* = 0.177, *R* ^2^ = 0.034, *b* = 0.225 (s.e. = 0.16), or among 6-year-olds, *F*(1,37) = 0.44, *p* = 0.513, *R* ^2^ = 0.012, *b* = −0.123 (s.e. = 0.19). Together, it appears that when deciding whether or not to believe a surprising claim, older children are more uncertain when they decide to believe the claim and more certain when they reject the claim, while younger children are most certain when they believe the claim and most uncertain when they reject the claim.

### 3.3. Do children express more plausible reasons for their uncertainty about the possibility of a claim with increasing age?

**Figure 4.**
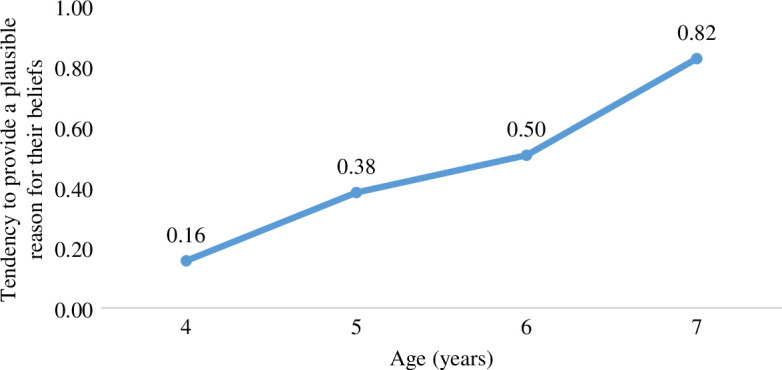
Children’s tendency to provide plausible explanations for their relative certainty in their beliefs across age groups.

To test Hypothesis 2 and the prediction that children provide more plausible explanations for their relative certainty in their beliefs, we asked children in the prompted condition *why* they believed what they did. As illustrated in figure 4, and confirmed by a linear regression with children’s Reasoning score as the dependent variable and Age (in years) as the independent variable, children provided more plausible explanations for their beliefs with increasing age, *F*(1,85) = 37.00, *p* < 0.001, *R* ^2^ = 0.303, *b* = 0.210 (s.e. = 0.03).

Control analyses revealed no effect of Block, *F*(1,85) = 0.30, *p* = 0.583, *R* ^2^ = 0.004, *b* = −0.048 (s.e. = 0.09); Order, *F*(1,85) = 2.55, *p* = 0.114, *R* ^2^ = 0.029, *b* = −0.138 (s.e. = 0.09); or Gender, *F*(1,85) = 0.21, *p* = 0.652, *R* ^2^ = 0.002, *b* = −0.040 (s.e. = 0.09), and including these control variables did not change the main model described above, Age always remained a significant predictor of children’s tendency to provide a plausible reason for their beliefs (all *p*s < 0.05) and there were no interactions (all *p*s > 0.05).

### 3.4. Are children more likely to suggest targeted empirical tests for a claim with increasing age? And does this effect hold also when controlling for children’s ability to select an efficient test

**Figure 5.**
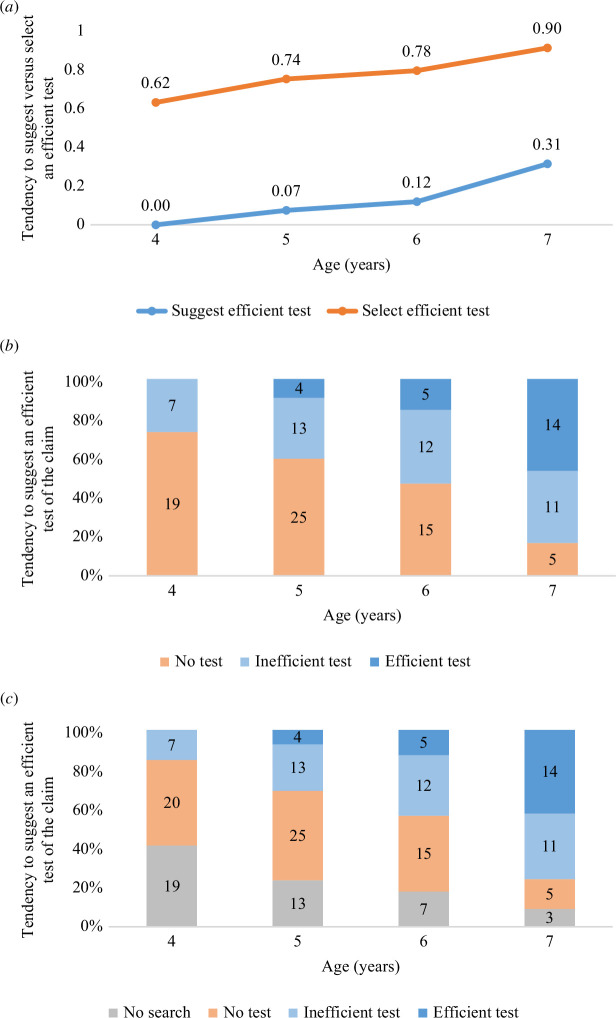
(*a*) Children’s tendency to suggest (blue line) versus select (orange line) an efficient, as opposed to inefficient, test of a claim, for each age group and condition, collapsing across the four trials. Note that a higher score reflects a greater probability of suggesting or selecting an efficient test (0 = Never suggested/selected an efficient test, 1 = Always suggested/selected an efficient test). (*b*) Number of children who wanted to find out the truth of a claim and either provide: no suggestion on how to test (orange bars), suggested only inefficient testing strategies (light blue bars), suggested an efficient search strategy on one or more occasions (dark blue bars), for each age group and collapsed across conditions and trials (i.e. as reflected in the *DesignAny* score). (*c*) Number of children who either did not want to find out the truth of the claim (grey bars) or wanted to find out the truth of a claim but either provided: no suggestion on how to test (orange bars), suggested only inefficient testing strategies (light blue bars), suggest an efficient search strategy on one or more occasions (dark blue bars), for each age group and collapsed across conditions and trials (i.e. as reflected in the *DesignAll* score).

Testing Hypothesis 3, and in line with our prediction, a linear regression with the *DesignSum* score as the dependent variable and Age (in years) as the independent variable showed a main effect of Age, *F*(1,64) = 13.44, *p* < 0.001, *R* ^2^ = 0.174, *b* = 0.432 (s.e. = 0.12) (see table 1, model 1). As illustrated in figure 5*a* (blue line), children become more likely to suggest an efficient test of a surprising claim with increasing age. A similar effect of Age was also found when using the *DesignAverage* score, *F*(1,64) = 10.72, *p* = 0.017, *R* ^2^ = 0.144, *b* = 0.126 (s.e. = 0.04); the *DesignAny* score, *F*(1,128) = 34.77, *p* < 0.001, *R* ^2^ *=* 0.214, *b* = 0.331 (s.e. = 0.06); and the *DesignAll* score, *F*(1,171) = 46.26, *p* < 0.001, *R* ^2^ *=* 0.213, *b* = 0.421 (s.e. = 0.06), as the dependent variables.

**Table 1.** Linear regression predicting children’s tendency to suggest an efficient test of a claim, as reflected in their *DesignSum* score and as a function of age and their ability to select an efficient test, as well as their interaction term.

	model 1	model 2	model 3
	*b* [LLCI, ULCI]	*b* [LLCI, ULCI]	*b* [LLCI, ULCI]
(intercept)	−1.89** [−3.30,−0.47]	−2.10** [−3.64,−0.57]	0.98 [−5.38, 7.34]
age	0.43** [0.20, 0.67]	0.39** [0.12, 0.65]	−0.18 [−1.34, 0.99]
select	—	0.19 [−0.33, 0.70]	−1.06 [−3.60, 1.49]
age by select	—	—	0.22 [−0.23, 0.68]
*R* ^2^	0.174**	0.180**	0.193**
Δ*R* ^2^	—	0.007	0.013
*N*	65	65	65
model d.f.	1	2	3

Note: *b* represents unstandardized regression weights. LLCI and ULCI indicate the lower and upper limits of a confidence interval, respectively. *p < 0.05. **p < 0.01.

To control for one potential explanation for the observed age change—children’s ability to identify an informative test—we asked all children to select the appropriate test for a given claim three times at the end of the testing session. In line with our expectation, and as illustrated in figure 5*a* (orange line), the majority of children were able to select an efficient test for the claim on at least one of the three occasions (*n* = 143; 82.18%), a tendency that increased with Age, *F*(1,172) = 38.49, *p* < 0.001, *R* ^2^ = 0.183, *b* = 0.260 (s.e. = 0.04). Furthermore, when introduced as the sole predictor of children’s tendency to suggest an efficient test, a linear regression revealed a significant main effect of Select, *F*(1,64) = 4.71, *p* = 0.034, *R* ^2^ = 0.069, *b* = 0.530 (s.e. = 0.24). Children who were better able to identify an efficient test, were also more likely to suggest one. However, including this as second step in the above model did not improve model fit, and age remained the only significant predictor (for details, see table 1, model 2). Furthermore, as illustrated in table 1, model 3, there was no Age by Select interaction (*p* > 0.05).

Control analyses revealed no effect of Uncertainty, *F*(1,64) = 0.96, *p* = 0.330, *R* ^2^ = 0.015, *b* = −0.464 (s.e. = 0.47); Block, *F*(1,64) = 1.54, *p* = 0.220, *R* ^2^ = 0.023, *b* = 0.328 (s.e. = 0.26); Order, *F*(1,64) = 0.28, *p* = 0.595, *R* ^2^ = 0.004, *b* = 0.142 (s.e. = 0.26); or Gender, *F*(1,64) = 1.37, *p* = 0.246, *R* ^2^ = 0.021, *b* = −0.313 (s.e. = 0.26). The effect of Age remained significant also when including these terms as preliminary steps in the model (all *p*s < 0.05) and there were no interactions (all *p*s > 0.05).

Note, however, that the sample size in the above analyses (table 1, models 1–3, *n* = 65) is smaller than we initially anticipated (estimated required *n* = 77–141 based on our *a priori* power analysis). The smaller sample size is in part due to children being less likely to want to find out the truth of the claim (roughly 74% of total sample, and not the anticipated 80%), but also due to a restricting coding plan, excluding children who wanted to explore the claim but did not specify a test (*n* = 56, 43.08%). As an alternative approach to improve statistical power and as part of our exploratory analyses, we thus re-ran the above analysis, but this time including all children who expressed an interest in finding out the truth of the claim, as reflected in the *DesignAny score* (*n* = 129). In that coding scheme, children who did not specify a test (i.e. saying nothing or ‘I don’t know’) were scored as not generating an efficient test rather than being excluded. This revealed, as before, a significant main effect of Age (for details, see table 2, model 1). This effect of age remained when introducing Select in the second step of the model (model 2), and there was no Age by Select interaction (model 3). Similarly, including all children using the *DesignAll score* as the dependent variable (*n* = 174), a score based on a coding scheme in which children who responded that they did not want to find out whether the claim was true or not were scored as not interested in testing rather than being excluded, again revealed a main effect of Age (for details, see table 3, model 1). Importantly, this effect remained also when introducing Select as a second step in the model (model 2), and there was no Age by Select interaction (model 3). Thus, in line with Hypothesis 3, age was a robust predictor of children’s inclination to efficiently test a surprising claim. It remained a significant predictor when controlling for children’s ability to identify an efficient test.

**Table 2.** Linear regression predicting children’s tendency to suggest an efficient test of a claim, as reflected in their *DesignAny* score and as a function of age and their ability to select an efficient test, as well as their interaction term.

	model 1	model 2	model 3
	*b* [LLCI, ULCI]	*b* [LLCI, ULCI]	*b* [LLCI, ULCI]
(intercept)	−1.14** [−1.76,−0.52]	−1.38** [−2.04,−0.72]	0.92 [−1.52, 3.37]
age	0.33** [0.22, 0.44]	0.28** [0.16, 0.40]	−0.16 [−0.62, 0.30]
select	—	0.23* [0.01, 0.45]	−0.76 [−1.79, 0.27]
age by select	—	—	0.18 [−0.00, 0.37]
*R* ^2^	0.214**	0.238**	0.260**
Δ*R* ^2^	—	0.025*	0.022
*N*	129	129	129
model d.f.	1	2	3

Note: *b* represents unstandardized regression weights. LLCI and ULCI indicate the lower and upper limits of a confidence interval, respectively. *p < 0.05. **p < 0.01.

**Table 3.** Linear regression predicting children’s tendency to suggest an efficient test of a claim, as reflected in their *DesignAll* score and as a function of age and their ability to select an efficient test, as well as their interaction term.

	model 1	model 2	model 3
	*b* [LLCI, ULCI]	*b* [LLCI, ULCI]	*b* [LLCI, ULCI]
(intercept)	−1.00** [−1.66,−0.33]	−1.25** [−1.93,−0.57]	0.56 [−1.83, 2.95]
age	0.42** [0.30, 0.55]	0.35** [0.21, 0.48]	−0.02 [0.50, 0.46]
select	—	0.30** [0.08, 0.51]	−0.48 [1.48, 0.53]
age by select	—		0.15 [−0.04, 0.35]
*R* ^2^	0.213**	0.244**	0.255**
Δ*R* ^2^	—	0.031**	0.011
*N*	173	173	173
model d.f*.*	1	2	3

Note: *b* represents unstandardized regression weights. LLCI and ULCI indicate the lower and upper limits of a confidence interval, respectively. **p < 0.01.

### 3.5. Does uncertainty reasoning increase the likelihood that children generate an efficient test for that claim, and does this change with age?

Testing Hypothesis 4, whether prompting children to reflect on their uncertainty would increase the probability that they suggest an efficient test for a surprising claim, and that this would be stronger for younger than older children, a linear regression with the *DesignSum* as the dependent variable, and Age (in years), Condition and their interaction term as the independent variables, revealed a main effect of Age, *F*(1,64) = 13.44, *p* < 0.001, *R* ^2^ = 0.174, *b* = 0.432 (s.e. = 0.11), but no main effect of Condition when adding this as a second step in the model, *F* ^Δ^(2,63) = 0.03, *p* = 0.853, *R^2^* ^Δ^ = −0.013, *b* = 0.021 (s.e. = 0.24), or any Age by Condition interaction, *F* ^Δ^(3,62) = 0.01, *p* = 0.971, *R* ^2Δ^ = −0.014, *b* = 0.026 (s.e. = 0.25). Controlling for children’s relative uncertainty in their belief, or their ability to select an efficient test did not change this outcome, Age always remained a significant predictor (all *p* < 0.05), and Condition never predicted children’s tendency to suggest an efficient test (all *p*s > 0.05).

However, although prompts did not have a direct effect on the informativeness of children’s testing, we wanted to explore whether the way in which children reasoned might. That is, whether being able to provide a plausible reason for their uncertainty, as reflected in their Reasoning score (i.e. whether they were able to reflect on their beliefs and provided a plausible reason for that belief), might affect children’s inclination to suggest an efficient test of a claim. Indeed, a linear regression with Reasoning introduced as a first step in the model, revealed a significant main effect, *F*(1,34) = 5.62, *p* = 0.024, *R* ^2^ = 0.142, *b* = 1.107 (s.e. = 0.47). However, when including Age as a second step in the model, only the effect of Age remained statistically significant (*p* < 0.05), and there was no Age by Reasoning interaction (*p* > 0.05) (for details, see table 4).

**Table 4.** Linear regression predicting children’s tendency to suggest an efficient test of a claim, as reflected in their *DesignSum* score and as a function of age, reasoning and their interaction term.

	model 1	model 2	model 3
	*b* [LLCI, ULCI]	*b* [LLCI, ULCI]	*b* [LLCI, ULCI]
(intercept)	−0.08 [−0.08, 0.64]	−1.80 [−3.44,−0.15]	−0.31 [−3.79, 3.17]
reasoning	1.11* [0.16, 2.06]	0.56 [−0.45, 1.58]	−1.76 [−6.65, 3.13]
age	—	0.36* [0.04, 0.67]	0.06* [−0.61, 0.74]
reasoning by age	—	—	0.43 [−0.46, 1.32]
*R^2^*	0.142*	0.263**	0.285*
Δ*R* ^2^	—	0.121*	0.022
*N*	35	35	35
model d.f.	1	2	3

Note: *b* represents unstandardized regression weights. *LLCI and ULCI i*ndicate the lower and upper limits of a confidence interval, respectively. *p < 0.05. **p < 0.01.

*Note* though that the sample size in the above analysis (*n* = 35) is far smaller than we initially anticipated for these analyses (estimated required *n* = 77–141 based on a prior power analysis). Thus, in an attempt to improve statistical power and as part of our exploratory analyses, we thus re-ran the above analysis, but this time including all children who expressed an interest in finding out the truth of the claim, as reflected in the *DesignAny* score (*n* = 65). That is, we included children who wanted to explore but failed to generate any test. This revealed, as before and as illustrated in figure 5*b*, a significant main effect of Reasoning (for details, see table 5, model 1). This now remained a significant predictor, also when including Age in the second step (model 2), as well as when controlling for children’s ability to Select an efficient test (model 3). Similarly, as illustrated in figure 5*c*, using the *DesignAll* score as the dependent variable (*n* = 86), including children who responded that they did not want to find out whether the claim was true and scored as ‘not interested in testing’ (0), we found a main effect of Reasoning (for details, see table 6, model 1), an effect that remained when including Age (model 2), as well as Select (model 3).

**Table 5.** Linear regression predicts children’s tendency to suggest an efficient test of a claim, as reflected in their *DesignAny* score and as a function of age, reasoning and select.

	model 1	model 2	model 3
	*b* [LLCI, ULCI]	*b* [LLCI, ULCI]	*b* [LLCI, ULCI]
(intercept)	0.24 [−0.03, 0.51]	−0.74 [−1.61, 0.14]	−0.92 [−1.86, 0.01]
reasoning	0.97** [0.56, 1.39]	0.72** [0.26, 1.18]	0.66** [0.19, 1.13]
age	—	0.20* [0.03, 0.37]	0.17 [−0.02, 0.35]
select	—	—	0.18 [−0.14, 0.50]
*R* ^2^	0.253**	0.313**	0.327**
Δ*R^2^*	—	0.060*	0.013
*N*	65	65	65
model d.f.	1	2	3

Note: *b* represents unstandardized regression weights. LLCI and ULCI indicate the lower and upper limits of a confidence interval, respectively. *p < 0.05. **p < 0.01.

**Table 6.** Linear regression predicting children’s tendency to suggest an efficient test of a claim, as reflected in their *DesignAll* score and as a function of age, reasoning and select.

	model 1	model 2	model 3
	*b* [LLCI, ULCI]	*b* [LLCI, ULCI]	*b* [LLCI, ULCI]
(intercept)	0.75** [0.48, 1.02]	−0.49 [−1.47, 0.50]	−0.67 [−1.72, 0.37]
reasoning	1.30** [0.85, 1.75]	0.92** [0.40, 1.45]	0.86** [0.32, 1.40]
age	—	0.26* [0.06, 0.46]	0.23* [0.02, 0.44]
select	—	—	0.18 [−0.16, 0.52]
*R^2^*	0.277**	0.331**	0.340**
Δ*R^2^*		0.054*	0.009
*N*	86	86	86
model d.f.	1	2	3

Note: *b* represents unstandardized regression weights. LLCI and ULCI indicate the lower and upper limits of a confidence interval, respectively. *p < 0.05. **p < 0.01.

Control analyses with *DesignAny* as the dependent variable revealed no effect of Block, *F*(1,128) = 1.80, *p* = 0.183, *R^2^* = 0.014, *b* = 0.178 (s.e. = 0.13); Order, *F*(1,128) = 0.07, *p* = 0.785, *R^2^* < .001, *b* = 0.036 (s.e. = 0.13); or Gender, *F*(1,128) = 0.02, *p* = 0.878, *R* ^2^ < 0.001, *b* = −0.021 (s.e. = 0.13). In all analyses, the effect of reasoning remained significant when introducing these control variables (all *p*s < 0.05), and there were no interactions (all *p*s > 0.05). Control analyses with *DesignAll* as the dependent variable revealed no effect of Order, *F*(1,172) = 0.08, *p* = 0.768, *R* ^2^ < 0.001, *b* = −0.044 (s.e. = 0.15); or Gender, *F*(1,172) = 1.56, *p* = 0.213, *R* ^2^ = 0.010, *b* = 0.019 (s.e. = 0.15). There was, however, an effect of Block, *F*(1,172) = 4.80, *p* = 0.030, *R* ^2^ = 0.029, *b* = 0.322 (s.e. = 0.15), with children in Block 2 being more likely to suggest an efficient test compared with children in Block 1. In all analyses, the effect of Reasoning remained significant when introducing these control variables (all *p*s < 0.05), and there were no interactions (all *p*s > 0.05).

Together with the planned analyses, this provides partial support for Hypothesis 4: Prompting children to provide an explanation for their beliefs did not directly affect their inclination to efficiently test the claim. However, when exploring children’s responses to these prompts and their explanations for their beliefs, we found that children who were able to provide a plausible reason for why they thought the way they did, were also more inclined to suggest an efficient test of the claim. Given that this effect of uncertainty reasoning held also when controlling for children’s ability to identify an efficient test, this provides convincing support for the notion that having the skills to reason scientifically (i.e. knowing how to generate a hypothesis and knowing how to isolate variables to test the hypothesis) is not sufficient to prompt children to follow up on their scepticism by efficiently testing what they have been told, they must also be able to understand why they are sceptical in the first place (i.e. knowing that they are uncertain about the truth of a claim because they lack particular evidence for that claim).

## 4. Discussion

Children become increasingly sceptical of surprising claims as they get older [28,40,41] and engage in more targeted and efficient exploration following surprising claims [4,31]. One potential mechanism for this development is that with increasing age children become better at knowing how to test various claims (i.e. an increase in scientific reasoning). An alternative mechanism supported by the current findings, is that older children’s more efficient testing of surprising claims reflects a greater awareness of *why* they are sceptical of those claims (i.e. an increase in metacognitive awareness). Below, we discuss the study’s four main hypotheses and the findings leading to this conclusion.

### 4.1. Children become increasingly sceptical of surprising claims, and certain about it, with age

In line with prior research, we found a significant and robust effect of age on children’s inclination to express scepticism towards a surprising claim [28,41]. Older children were more likely to reject the claim, compared with younger children. However, in contrast to our initial prediction for Hypothesis 1, this increasing scepticism was not paired with increased uncertainty about what to believe. At first glance, older children appeared more certain about their beliefs than younger children. However, our exploratory analysis revealed that 7-year-old children were most certain when rejecting the adult’s surprising claim and most uncertain when accepting it, while 4-year-old children were more certain when accepting the claim and more uncertain when rejecting it.

To understand this pattern, it is worth reflecting on how children’s evaluations of informants and their claims change with age: with increasing age, children become more discerning about when adults have relevant expertise, increasingly confident about the information they possess, and better at weighing conflicting evidence. For instance, from around age 5, children become more sceptical of surprising claims when they can compare those claims to perceptual [40] or physical evidence [29], and from around 7 years of age children are more likely to trust an expert informant rather than their mother [42]. However, 5- and 8-year-old children are still developing their ability to evaluate inconclusive evidence [43] and become better at determining when to update their beliefs based on the evidence they possess [44]. By implication, in our study, 4-year-old children may have been less certain about their own belief *and* more likely to have considered the informant as knowledgeable than 7-year-old children. As a result, 4-year-old children tended to confidently endorse the informant’s claims. In contrast, 7-year-old children tended to confidently reject those claims. As we deliberate on in the section below, one potential explanation for this pattern may be that older children are better able to reflect on the intuitions guiding their decision to accept or reject the claim.

### 4.2. Children provide more plausible reasons for their beliefs as they get older

Supporting Hypothesis 2, there was a clear effect of age on children’s ability to provide a plausible, as opposed to not plausible, explanation for their belief. Regardless of whether they were certain or uncertain about the adult’s surprising claim, children were better able to explicitly reflect on their reasons for their beliefs with increasing age. This fits well with prior work showing that children between 4 and 7 years of age improve in their ability to engage in diagnostic reasoning [18], an ability that develops independently of their ability to evaluate evidence [45]. It also resonates with the notion that children during this period undergo a transition from thinking with their theories to thinking about them [46]. As we discuss in further detail below, it also aligns with past works suggesting that children’s greater metacognitive awareness about the reasons for their beliefs may support their ability to seek out evidence that can distinguish between opposing claims.

### 4.3. Children become more likely to suggest an efficient test of a claim with increasing age

Two findings were essential to support Hypothesis 3 and the prediction that, with age children become increasingly likely to suggest an efficient test of a surprising claim, controlling for their ability to identify such a test. First, the analyses revealed a clear effect of age on children’s inclination to suggest an efficient test for the claim when asked whether they would like to find out the truth of a claim, and in turn how they would propose to do so. Older children were more likely to suggest an efficient test compared with younger children. This finding extends prior work by showing an age change in children’s inclination to engage in targeted testing of surprising claims not only in a physical lab environment [4,31] but also on an online platform. Second, more importantly, although children showed a significant improvement in scientific reasoning with increasing age, as indexed by their ability to identify an efficient test of a claim when presented with a set of options, this did not explain age-related improvements in children’s inclination to suggest such a test when left to their own devices.

### 4.4. Uncertainty reasoning explains children’s ability to design an efficient test of a claim

To explore the role of metacognitive processes on children’s developing critical stance, and because explanations are likely to aid hypothesis generation [47], we prompted half of the children to reason about the causes of their relative (un)certainty in their beliefs about the adult’s claim. The other half of the children received no such prompt. In contrast to our initial prediction for Hypothesis 4, that prompting children to reason about their uncertainty would be associated with more suggestions as to how to efficiently test a claim, and that younger children in particular would benefit from such prompts, our initial analytical plan did not support this conclusion. One reason why we did not see a condition effect is that younger children appear to lack the capacity to introspect on the reasons for their uncertainty. Unlike older children, they frequently failed to provide any reasons and when they did provide reasons these reasons were often coded as not plausible. Thus, our experimental manipulation of prompting revealed that younger children fail to test claims in an efficient manner not because they fail to reflect on their beliefs but instead because they lack the capacity to do so. The pattern we observed also supports the notion that not all (self-)explanations are helpful [48]. Which is not a trivial matter, because as Kuhn and Katz suggest ‘…when children repeatedly explain their preexisting theories (…), they become more committed to them’ [48, p.393], and future research (as well as pedagogical interventions) aimed at improving the efficiency by which children process and investigate surprising information through the use of explanations should pay careful attention to the way in which children generate their explanations and the kinds of explanations they generate.

Of note, our exploratory analyses using a more inclusive coding scheme, revealed that children’s ability to provide an explanation for their beliefs predicted their tendency to suggest an efficient test. Critically, this effect held when controlling for children’s capacity to select the most informative test—an important aspect of scientific reasoning. This set of results is consistent with the notion that explanations drive children’s exploratory behaviours [34] and with past work showing that children as young as 4 years of age are able to select the most informative interventions and questions when presented with a causal system that does not rely on prior content knowledge [2,21,35]. It is also consistent with past work showing that children’s ability to engage in diagnostic reasoning about uncertain causes develops during this same time period [18].

Why does the ability to reflect on and justify one’s beliefs but not the ability to select an informative test predict children’s ability to come up with a more efficient test for a claim on their own? We propose the following explanation. When children are asked to select the best question to solve a problem or to select the best intervention for a simple causal system, children are essentially presented with a restricted set of possible questions and interventions. This restricted hypothesis space scaffolds their ability to identify the most efficient question or intervention. For example, in Lapidow *et al*. [11], when children are given three alternatives as to what they would like to explore, their exploration is driven by what is objectively more uncertain, but not by their subjective reports of uncertainty. When children are presented with a more complex problem, as in the current study, children’s ability to form and justify a hypothesis predicts their ability to design an efficient test. Reflecting on one’s beliefs narrows the hypothesis space one needs to consider and makes it possible to select the right intervention or question. The fact that metacognition—thinking about thinking—is related to children’s ability to test a surprising claim is consistent with longitudinal data on children’s scientific reasoning (which did not test children’s hypothesis generation) that shows that recursive theory of mind—the understanding that the beliefs are malleable and constantly changing—is a driver of preschoolers’ emergent scientific thinking (and not vice versa), while interpretative theory of mind—the understanding that changing beliefs have a cause—is central to older elementary school children’s scientific thinking capacities [49]. As such, these findings bridge the gap between the literature on children’s early intuitive and informative exploratory behaviours and their later strategic and scientific stance, by highlighting the importance of being able to reflect on one’s belief for children’s ability to efficiently test a claim.

### 4.5. Limitations and future directions

A key aim of this study was to assess whether children would suggest more targeted empirical testing of a claim when prompted to explain their beliefs. However, while our planned analyses did not show an effect of merely prompting children to reflect on their beliefs, our exploratory analyses showed a clear effect of the *kind* of explanations children came up with. Children who provided more plausible reasons for their beliefs suggested more targeted testing of the claim. Motivated by the lower-than-expected sample size for these analyses, we found that this result was robust across multiple coding schemes. However, given the exploratory nature of these analyses, future research should test this association directly. Nonetheless, these results highlight that not all explanations are good explanations and that there is pedagogical potential in developing interventions to aid children’s ability to reason about why they believe what they believe—possibly starting before and outside of formal educational contexts.

When designing our study, we were careful not to present the informant as particularly knowledgeable, given prior work showing that young children are more likely to defer when their informant is knowledgeable because they ‘know’ (as opposed to ‘think’) something [50] or because they are explicitly labelled as knowledgeable [51] or expert [52]. However, our use of adult informants rather than peer informants could have disproportionally influenced younger children’s trust in the testimony they were given. Indeed, 3- to 5-year-old children maintain their beliefs in moral or visual domains when faced with peers rather than adults presenting a conflicting claim [53]. This suggests that future research might examine whether engaging young children in tasks where they must explicitly reason about the cause of their beliefs in the presence of, or together with, a child rather than an adult informant may support their reasoning and empirical testing. It could lead 4-year-old children (who in our study were most uncertain when questioning the adult’s claim) to be more likely to openly and critically reflect on the causes of their beliefs.

## 5. Conclusion

The essential contribution of the current study is that it highlights the connection between children’s ability to reflect on their uncertainty and their ability to design an informative test of a claim. Based on data from a pre-registered experimental design, confirmatory and exploratory analysis revealed that children become increasingly sceptical of surprising claims with age, a scepticism they become more certain about, and better able to provide explanations for, as they get older. Children also become more likely to suggest an efficient test of a claim with increasing age. Importantly, this age change in the efficiency of children’s investigative behaviours is best predicted by children’s ability to provide reasons for their beliefs. As such, the present findings advance our understanding of children’s transition from intuitive science [5] to explicit scientific thinking [6] and highlight the role of metacognition in that transition.

## Data Availability

Due to the privacy of the research participants, the video recordings of the participants will not be made publicly available. However, anonymized data supporting the findings of this study will be available in the Open Science Framework at [37]. The manuscript was accepted following stages 1 and 2 reviews at Peer Community In Registered Reports and recommendations can be viewed at [38].

## Appendix A

See table 7.

**Table 7.** Proforma study design template.

Question	Hypothesis	Sampling plan	Analysis Plan	Rationale for deciding the sensitivity of the test for confirming or disconfirming the hypothesis	Interpretation given different outcomes	Theory that could be shown wrong by the outcomes	Observed outcome
Q1: Do children express more uncertainty about the possibility of a surprising claim with increasing age?	H1: With increasing age, children will express more uncertainty about the possibility of a surprising claim.	We base our sampling plan on a power calculation, using GPower 3.1. [36], indicating a need for ca. 175 participants to meet the criteria of the most resource demanding analyses.	Linear regression — Dataset: Full — Dependent variable: Uncertainty_Average — Independent variable: Age	Effect size estimates used in the power analyses are derived from previous studies with a similar design and age range indicating that we should expect low-medium to medium effects (f2 = .08-.15)[ 4,30,31).We use standard norms to set alpha = .05 and power = 80%.	H1 is supported if the effect of Age is significant below the threshold of alpha = .05.If Age is not a significant predictor, this questions the age change observed in prior studies showing an increased sensitivity to the uncertainty of surprising claims.	Older children do not express increased rates of uncertainty about the possibility of a surprising claim compared to younger children.	Confirmatory analysis not supported. Exploratory analyses taking into account children’s belief in the claim show that older children are more uncertain when trusting the claim, while younger children are more uncertain when questioning the claim.
Q2: Do children express more plausible reasons for their uncertainty about the possibility of a claim with increasing age?	H2: With increasing age, children are more likely to provide a plausible reason for their uncertainty about the possibility of a surprising claim.		Linear regression — Dataset: Prompted condition only — Dependent variable: Reasoning_Average — Independent variable: Age		H2 is supported if the effect of Age is significant below the threshold of alpha = .05.If Age is not a significant predictor, this questions the age change observed in prior studies showing improvements in their ability to express reasons for their uncertainty.		Confirmatory analyses supported.
Q3: Are children more likely to suggest targeted empirical tests for a claim with increasing age?And does this effect hold also when controlling for children’s ability to select an efficient test?	H3: Children are more likely to suggest targeted empirical tests for a claim with increasing age.And this is not driven by their ability to select an efficient test.		Linear regression I — Dataset: Full — Dependent variable: Design_Sum — Control variable: Condition — Independent variable: Age — Covariates: Uncertainty_Average, Selection_Average Linear regression II — Dataset: Full — Dependent variable: Design_Average — Control variable: Condition — Independent variable: Age — Covariates: Uncertainty_Average, Selection_Average		H3 is supported if the effect of Age is significant below the threshold of alpha = .05.If Age is not a significant predictor, this questions the age change observed in prior studies showing that older children are more likely to test or suggest a test for a surprising claim.	Prompts to uncertainty alone does not increase younger children’s testing of a surprising claim.	Confirmatory analyses supported.
Q4: Does prompting children to reflect on their uncertainty increase the likelihood that they generate an efficient test for that claim? And is this effect stronger for younger than older children?And do these effects hold also when controlling for children’s ability to select an efficient test?	H4: Prompting children to reflect on the causes of their uncertainty about a claim will increase the likelihood that they generate an efficient test of that claim. This effect of prompting is expected to be stronger among younger children.And these effects are not driven by their ability to select an efficient test.		Linear regression I — Dataset: Full — Dependent variable: Design_Sum — Independent variables: Condition, Age, Condition BY Age — Covariate: Selection_Average Linear regression II — Dataset: Full — Dependent variable: Design_Average — Independent variables: Condition, Age, Condition BY Age — Covariate: Selection_Average		H4 is supported if the main effect of Condition is significant below the threshold of alpha = .05, and/or there is a significant Condition BY Age interaction.If there is only an effect of Age, this suggest that reasoning about the causes of one’s uncertainty may not be the key driver of the propensity to test the veracity of a claim. If the there is only a significant effect of the covariate reflecting children’s ability to select the most efficient test, this suggest that scientific reasoning may be a key driver of children’s inclination to test a claim.	Prompts to reason about their uncertainty about surprising claims does not increase younger children’s inclination to suggest an efficient test any more than older children.Scientific reasoning is not key driver of children’s inclination to test a claim.	Confirmatory analyses not supported. Exploratory analyses taking into account the type of explanation children provide when prompted showed that when able to provide a plausible reason for their beliefs, children were more likely to generate an efficient test for that claim.
